# Education as Risk Factor of Mild Cognitive Impairment: The Link to the Gut Microbiome

**DOI:** 10.14283/jpad.2024.19

**Published:** 2024-01-23

**Authors:** Matthias Klee, V. T. E. Aho, P. May, A. Heintz-Buschart, Z. Landoulsi, S. R. Jónsdóttir, C. Pauly, L. Pavelka, L. Delacour, A. Kaysen, R. Krüger, P. Wilmes, A. K. Leist, NCER-PD Consortium, Geeta Acharya, Gloria Aguayo, Myriam Alexandre, Muhammad Ali, Wim Ammerlann, Giuseppe Arena, Michele Bassis, Roxane Batutu, Katy Beaumont, Sibylle Béchet, Guy Berchem, Alexandre Bisdorff, Ibrahim Boussaad, David Bouvier, Lorieza Castillo, Gessica Contesotto, Nancy De Bremaeker, Brian Dewitt, Nico Diederich, Rene Dondelinger, Nancy E Ramia, Angelo Ferrari, Katrin Frauenknecht, Joëlle Fritz, Carlos Gamio, Manon Gantenbein, Piotr Gawron, Laura Georges, Soumyabrata Ghosh, Marijus Giraitis, Enrico Glaab, Martine Goergen, Elisa Gómez De Lope, Jérôme Graas, Mariella Graziano, Valentin Groues, Anne Grünewald, Gaël Hammot, Anne-Marie Hanff, Linda Hansen, Michael Heneka, Estelle Henry, Margaux Henry, Sylvia Herbrink, Sascha Herzinger, Alexander Hundt, Nadine Jacoby, Sonja Jónsdóttir, Jochen Klucken, Olga Kofanova, Rejko Krüger, Pauline Lambert, Zied Landoulsi, Roseline Lentz, Laura Longhino, Ana Festas Lopes, Victoria Lorentz, Tainá M. Marques, Guilherme Marques, Patricia Martins Conde, Patrick May, Deborah Mcintyre, Chouaib Mediouni, Francoise Meisch, Alexia Mendibide, Myriam Menster, Maura Minelli, Michel Mittelbronn, Saïda Mtimet, Maeva Munsch, Romain Nati, Ulf Nehrbass, Sarah Nickels, Beatrice Nicolai, Jean-Paul Nicolay, Fozia Noor, Clarissa P. C. Gomes, Sinthuja Pachchek, Claire Pauly, Laure Pauly, Lukas Pavelka, Magali Perquin, Achilleas Pexaras, Armin Rauschenberger, Rajesh Rawal, Dheeraj Reddy Bobbili, Lucie Remark, Ilsé Richard, Olivia Roland, Kirsten Roomp, Eduardo Rosales, Stefano Sapienza, Venkata Satagopam, Sabine Schmitz, Reinhard Schneider, Jens Schwamborn, Raquel Severino, Amir Sharify, Ruxandra Soare, Ekaterina Soboleva, Kate Sokolowska, Maud Theresine, Hermann Thien, Elodie Thiry, Rebecca Ting Jiin Loo, Johanna Trouet, Olena Tsurkalenko, Michel Vaillant, Carlos Vega, Liliana Vilas Boas, Paul Wilmes, Evi Wollscheid-Lengeling, Gelani Zelimkhanov

**Affiliations:** 1 Institute for Research on Socio-Economic Inequality (IRSEI), Department of Social Sciences, University of Luxembourg, 2, avenue de l’Université, L-4365 Esch-sur-Alzette, Luxembourg; 2 Luxembourg Centre for Systems Biomedicine, University of Luxembourg, 7, avenue des Hauts-Fourneaux, L-4362 Esch-sur-Alzette, Luxembourg; 3 Swammerdam Institute of Life Sciences at University of Amsterdam, Sciencepark 904, 1098 XH Amsterdam, the Netherlands; 4 Parkinson’s Research Clinic, Centre Hospitalier de Luxembourg, 4, rue Ernest Barblé, L-1210 Luxembourg (Belair), Luxembourg; 5 Transversal Translational Medicine, Luxembourg Institute of Health, 1A-B, rue Thomas Edison, L-1445 Strassen, Luxembourg; 6 Department of Neurology, Centre Hospitalier de Luxembourg, 4, rue Ernest Barblé, L-1210 Luxembourg (Belair), Luxembourg; 7 Department of Life Sciences and Medicine, Faculty of Science, Technology and Medicine, University of Luxembourg, 7, avenue des Hauts-Fourneaux, L-4362 Esch-sur-Alzette, Luxembourg; 8 Institute for Advanced Studies, University of Luxembourg, 2, avenue de l’Université, L-4365 Esch-sur-Alzette, Luxembourg; 9 Institute for Research on Socio-Economic Inequality, Department of Social Sciences, University of Luxembourg, 11, Porte des Sciences, L-4366 Esch-sur-Alzett, Luxembourg; 10 Luxembourg Centre for Systems Biomedicine, University of Luxembourg, Esch-sur-Alzette, Luxembourg; 11 Luxembourg Institute of Health, Strassen, Luxembourg; 12 Centre Hospitalier de Luxembourg, Strassen, Luxembourg; 13 Laboratoire National de Santé, Dudelange, Luxembourg; 14 Centre Hospitalier Emile Mayrisch, Esch-sur-Alzette, Luxembourg; 15 Parkinson Luxembourg Association, Leudelange, Luxembourg; 16 Association of Physiotherapists in Parkinson’s Disease Europe, Esch-sur-Alzette, Luxembourg, Luxembourg; 17 Private practice, Ettelbruck, Luxembourg; 18 Private practice, Luxembourg, Luxembourg; 19 Faculty of Science, Technology and Medicine, University of Luxembourg, Esch-sur-Alzette, Luxembourg; 20 Department of Epidemiology, CAPHRI School for Public Health and Primary Care, Maastricht University Medical Centre+, Maastricht, the Netherlands; 21 Luxembourg Center of Neuropathology, Dudelange, Luxembourg; 22 Department of Life Sciences and Medicine, University of Luxembourg, Esch-sur-Alzette, Luxembourg

**Keywords:** Dementia, mild cognitive impairment, gut microbiome, education, mediation

## Abstract

**Background:**

With differences apparent in the gut microbiome in mild cognitive impairment (MCI) and dementia, and risk factors of dementia linked to alterations of the gut microbiome, the question remains if gut microbiome characteristics may mediate associations of education with MCI.

**Objectives:**

We sought to examine potential mediation of the association of education and MCI by gut microbiome diversity or composition.

**Design:**

Cross-sectional study.

**Setting:**

Luxembourg, the Greater Region (surrounding areas in Belgium, France, Germany).

**Participants:**

Control participants of the Luxembourg Parkinson’s Study.

**Measurements:**

Gut microbiome composition, ascertained with 16S rRNA gene amplicon sequencing. Differential abundance, assessed across education groups (0–10, 11–16, 16+ years of education). Alpha diversity (Chao1, Shannon and inverse Simpson indices). Mediation analysis with effect decomposition was conducted with education as exposure, MCI as outcome and gut microbiome metrics as mediators.

**Results:**

After exclusion of participants below 50, or with missing data, n=258 participants (n=58 MCI) were included (M [SD] Age=64.6 [8.3] years). Higher education (16+ years) was associated with MCI (Odds ratio natural direct effect=0.35 [95% CI 0.15–0.81]. *Streptococcus* and *Lachnospiraceae*-UCG-001 genera were more abundant in higher education.

**Conclusions:**

Education is associated with gut microbiome composition and MCI risk without clear evidence for mediation. However, our results suggest signatures of the gut microbiome that have been identified previously in AD and MCI to be reflected in lower education and suggest education as important covariate in microbiome studies.

**Electronic Supplementary Material:**

Supplementary material is available for this article at 10.14283/jpad.2024.19 and is accessible for authorized users.

## Introduction

**M**odifiable social and behavioral risk factors of Alzheimer’s disease (AD) and related dementias convey potential to delay or prevent a substantial rate of cases, if targeted effectively (1). This entails interventions to be delivered early in the disease trajectory, informed by knowledge on working mechanisms. With respect to timeliness, research on biomarkers suggests AD-related pre-clinical pathophysiological changes occurring as early as midlife (2). At a later stage, mild cognitive impairment (MCI) reflects early, subtle changes in thinking and memory (3). While potentially due to a variety of underlying diseases or disorders, MCI is a markedly strong risk factor for AD. Furthermore, synergies in risk factors of MCI and AD exist, i.e., related to education and consequentially lifestyle (3).

Education itself reflects a well-established early-life risk factor for AD. As such, higher education is associated to lower dementia risk in later life (1). Lower dementia risk may result from education increasing cognitive abilities in early adulthood and consequent build-up of cognitive reserve, brain reserve or brain maintenance, protecting against neurodegeneration (1, 4). Moreover, risk factors such as obesity or smoking frequently cooccur, and vary in prevalence according to socioeconomic status (SES) (3, 5). Higher exposure to lifestyle-related risk factors according to education, an indicator of SES, may further contribute to a vascular pathway linking education to dementia risk. To date, there is no consensus about working mechanisms. However, recent studies suggest education, which influences life histories and in part constitutes SES, to be associated with differences in microbial community types across multiple body sites, which may be in turn associated with MCI risk (6–9).

The gut microbiome refers to a collection of microbes within the gastrointestinal tract (GIT). The GIT, reflecting the largest ecosystem of the human body, is composed of bacteria, archaea, eukaryotes, and other microbes. Composition of the gut microbiome, for instance differential abundance of specific taxa, is subject to interindividual variation, e.g., across the life-course or geographical locations (10). Factors affecting the microbiome over a lifetime are for instance linked to SES and early childhood conditions (e.g., mode of delivery, breast feeding) resulting in variation in consequent gut colonization and microbiome maturation, which may in turn continue to affect microbiome composition in later life (10-14).

Gut microbiome alterations have been observed, e.g., associated with ageing, or health. As such, ageing-related changes may result from the ageing processes (changing hormonal levels), changing health conditions (associated use of medication) or age-related behavioral changes (dietary deficiency) (10). Moreover, recent findings suggest a link of the gut microbiome to MCI or AD, and lifestyle-related risk factors such as diet or physical activity (7, 15–17). Potential working mechanisms along the gut-brain-axis likely involve complex pathways, e.g. triggering low-grade systemic inflammation by altering gut permeability or by synthesis of metabolites with neuroendocrine functions (18). Due to the likely involvement of specific molecules, the low resolution of marker gene-based microbiome analyses precludes further specification of molecular pathways.

The association of education to gut microbiome alterations and MCI risk motivate the investigation of the role of the gut microbiome in the relationship of education and MCI. Thus, we sought to examine potential mediation of the association of education and MCI by the gut microbiome in the present study.

## Methods

### Study Participants and Design

We analyzed data of participants, specifically the control subjects, from the Luxembourg Parkinson’s Study (LUXPARK) of the National Centre of Excellence in Research on Parkinson’s disease (NCER-PD), which received approval from the National Ethics Board (CNER Ref: 201407/13) and Data Protection Committee (CNPD Ref: 446/2017) and was conducted according to the Declaration of Helsinki (19). Eligibility criteria for analysis were age above 50, absence of Parkinson’s disease, celiac disease, and chronic inflammatory bowel disease, availability of stool samples and non-missing data. All participants provided written informed consent.

### 16S rRNA Gene Amplicon Sequencing Analysis

NCER-PD participants collected stool samples at home and sent them to the Integrated Biobank of Luxembourg (20). Sampling, processing, and sequencing of NCER-PD LUXPARK stool samples were done as previously described (20, 21). The 16S rRNA gene amplicon sequencing data was processed using the dadasnake workflow, a Snakemake pipeline to process amplicon sequencing data, based on DADA2 (22–24). Amplification primers were removed using cutadapt, allowing 20% mismatches and no indels (25). Quality filtering, amplicon sequence variant (ASV) generation and chimera removal were performed in DADA2. Reads were truncated at positions with less than 10 Phred score quality, or at 240 bp. The quality filtering kept only sequences with a maximum expected error of 2 and 240 bp length. Downsampling was performed to 25,000 reads using seqtk (https://github.com/lh3/seqtk:RRID:SCR_018927) and samples with smaller library sizes were removed from the downstream analysis. ASVs were generated in pooled mode for the whole study using DADA2 default parameters. For merging forward and reverse ASVs, a minimum overlap of 12 bp was required. Chimeric sequences were removed based on the consensus algorithm. Taxonomic classification was performed against SILVA v138 using the naïve Bayesian classifier implemented in mothur (26, 27). NCER-PD clinical and 16S rRNA gene amplicon sequencing data are available on request from https://www.parkinson.lu/research-participation.

### Main Exposures and Outcomes

Clinical assessments were conducted by neurologists, neuropsychologists, or trained study nurses. MCI classification was based on the Montreal Cognitive Assessment (MoCA), a brief measure for assessing cognitive function (28). MoCA scores below 26 led to MCI classification (28).

Education was assessed in years (YEDU). For analysis, YEDU were grouped (0–10 [reference], 10–16, 16+ YEDu) based on the ISCED classification scheme, group sizes, and differences in compulsory schooling duration in Luxembourg for participants of different age (29, 30).

Alpha diversity captures the diversity of the microbiome within individuals. Alpha diversity will be greater in individuals with a greater number of different taxa (= richness) and/or similar abundances of prevalent taxa (= evenness). Alpha diversity is subject to variation over the life-course and higher alpha diversity has been related to better health in older age (10, 16). Three measures for alpha diversity were computed after rarefication: Chao1, Shannon and inverse Simpson (Supplementary Material). Beta diversity reflects differences of the microbiome between individuals. In that, dissimilarity indices reflect pairwise distances between individuals based on taxa abundance. In a sample-by-sample distance matrix, a greater value in a given cell indicates a larger dissimilarity between two individuals. This information can be used to compare similarity of variance and composition of the gut microbiome between groups of individuals. Two measures for beta diversity were computed: Bray-Curtis dissimilarity and Jaccard distance (Supplementary Material).

### Covariates

Additional measures included sociodemographic indicators age, sex/gender, first language (French/ Luxembourgish/German versus other), partnership status (PS; married/domestic partnership versus widowed/never married/divorced/separated), body mass index (BMI), mild depressive symptoms based on the Beck Depression Inventory I (BDI-I; >9), use of antibiotic medication in the last 6 months (ATB; yes versus no), and apolipoprotein ε4 status (APOE; at least one versus no ε4 allele) (31).

### Statistical Analysis

All analyses were performed in R version 4.2.0 (Supplementary Material) (32). Analysis code is available online (https://github.com/makleelux/edu_biome_mci). Differences of descriptive characteristics in presence or absence of MCI were tested with Fisher’s Exact Test for categorical and Student’s t-Test for continuous characteristics. Differences in beta diversity were tested across education groups with betadisper [vegan] and adonis2 [vegan] with 999 permutations. In short, betadisper compares average distances, i.e. the dispersion or homogeneity, across groups, while adonis2 tests multivariate differences in microbiome compositions (33).

Differential abundance analysis (DAA) was conducted across education groups, adjusting for age, sex/gender, BMI, and ATB. DAA was repeated additionally adjusting for first language, PS, BDI-I, and APOE, as robustness check. Two commonly used functions were employed (ancombc [ANCOMBC]; DESeq [DESeq 2: RRID:SCR_000154]) (34, 35). Both methods identify differentially abundant taxa with estimates of statistical significance adjusted for false discovery rates (Supplementary Material). For DAA, taxa with nonzero counts in less than 25% of samples were not tested.

Mediation analysis was specified with MCI as outcome and groups of education as exposure, adjusting for age, sex/gender, first language, PS, BDI-I, APOE and ATB. For alpha diversity as mediator, a regression-based, counterfactual approach to mediation was employed for which continuous mediator models (i.e., alpha diversity as outcome) and a logistic outcome model (i.e., MCI as outcome), including interaction terms for education and alpha diversity, were specified (Supplementary Material; cmest [CMAverse]) (36). Total effects of education on MCI were decomposed into a controlled direct effect (CDE) for alpha diversity fixed at the sample mean, a natural direct effect (NDE, Supplementary Material), and a natural indirect effect (NIE, Supplementary Material) (37–39). Proportion eliminated (PE) was calculated, indicating the proportion of the effect due to either mediation, interaction, or both, that would be eliminated by fixing the mediator to a specific level, i.e., the sample mean of the z-standardized alpha diversity measures (38). As a sensitivity check, mediation analysis was repeated without interaction terms in the outcome model.

For beta diversity as mediator, a previously described inverse-regression-based approach to mediation was employed at genus level (40, 41). In short, this approach specifies regressions for potentially mediating taxa at genus level on education, and MCI adjusted for education, in turn utilizing resulting p values to test mediation. Two functions were used, allowing to estimate mediation by abundance of specific taxa or by the overall composition of the microbiome (ldm [LDM]; permanovaFL [LDM]), while controlling for false discovery rates (40–42). Ldm suggests mediation if education affects the microbiome and consequentially the outcome. This can be tested globally (community contains any mediating taxa) and locally (mediation by specific taxa). PermanovaFL is a distance-based procedure, and suggests mediation if education affects some part of the community and some potentially different part of the community proceeds to affect MCI, thus being less conservative. For ldm an omnibus test was conducted combining analysis at three scales (i.e., relative abundance, arcsin-root transformed relative-abundance, presence-absence) (43). For permanovaFL individual and omnibus tests were conducted combining analysis at two scales (i.e., relative abundance, presence-absence) (43).

## Results

### Participant Characteristics

From n=524 participants of the LUXPARK study without Parkinson’s disease or Parkinsonism diagnosis, n=258 participants were eligible for analysis (M [SD] Age=64.6 [8.3] years) after exclusion of participants below age 50 (n=93), with celiac disease (n=6) or chronic inflammatory bowel disease (n=5), missing data (n=11) or without stool samples and microbiome data (n=149, and n=2 after pruning of samples with library size <10,000). Participants with MCI (n=58) were older, more likely male, had fewer YEDU and a higher BMI (Table 1). A total of 1,150 taxa at seven taxonomic ranks were identified after trimming of ASVs occurring in <10% of samples and pruning of samples with library size <10,000.

**Table 1:** Baseline Characteristics of Participants

	**NC (n=200)**	**MCI (n=58)**	**P Value**	**Test**
Age, years	63.76 ± 7.84	67.6 ± 9	.005	t
BMI	27.04 ± 4.37	29.74 ± 5.91	.002	t
Sex/gender				
Female	87	15	.022	Fisher
Male	113	43		
Years of Education				
0–10	24	16	.018	Fisher
11–16	110	24		
16+	66	18		
First Language				
FR / LU / DE	182	52	.798	Fisher
Other	18	6		
Living With Partner				
No	61	17	1	Fisher
Yes	139	41		
BDI-I (>9)				
Yes	23	9	.497	Fisher
No	177	49		
APOE				
At least one ε4	54	19	.410	Fisher
No ε4	146	39		
Antibiotics (Last 6 Months)				
No	179	49	.351	Fisher
Yes	21	9		
Alpha Diversity				
Chao1	311.56 ± 64.96	295.05 ± 86.37	.181	t
Shannon	3.99 ± 0.38	3.93 ± 0.46	.415	t
Inverse Simpson	27.75 ± 11.46	26.79 ± 11.66	.580	t

Note. T = Student’s t-Test, Fisher = Fisher’s Exact Test. Numbers refer to means ± standard deviations for continuous, n for categorical characteristics. BMI = body mass index, BDI-I = Beck Depression Inventory I, APOE = apolipoprotein ε4 status.

**Figure 1:**
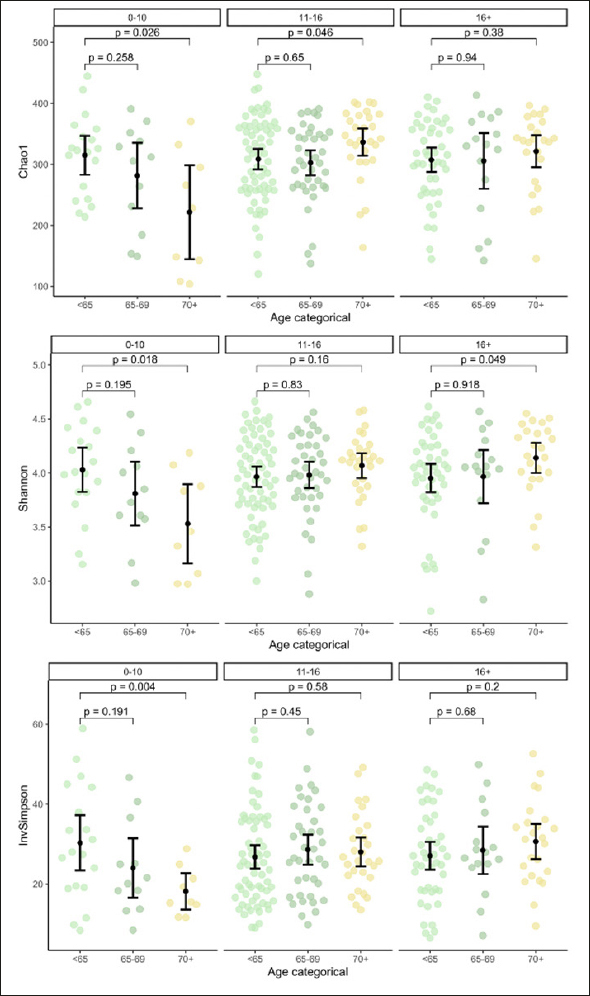
Alpha Diversity Across Age and Education Groups Note. Panels show alpha diversity stratified by age and education groups with 0–10, 11–16 and 16+ years of education. Reported P values refer to Student’s t-Tests. InvSimpson = Inverse Simpson. Author MK.

Alpha diversity as per Chao1 was lower in but not significantly associated with MCI (Table 1). Education groups did not differ significantly in beta dispersion, tested with anova (p=.17), thus meeting the assumption of homogeneity of variances for adonis2. Education groups did not differ significantly regarding multivariate analyses with adonis2 (p=.20; adjusting for sex/gender, age, ATB, BDI-I, first language, PS, and APOE), suggesting similar composition of the microbiome. However, alpha diversity was lower in lower education (Supplementary Figure 1) and was significantly lower in older age but only in lower education (Figure 1).

**Figure 2:**
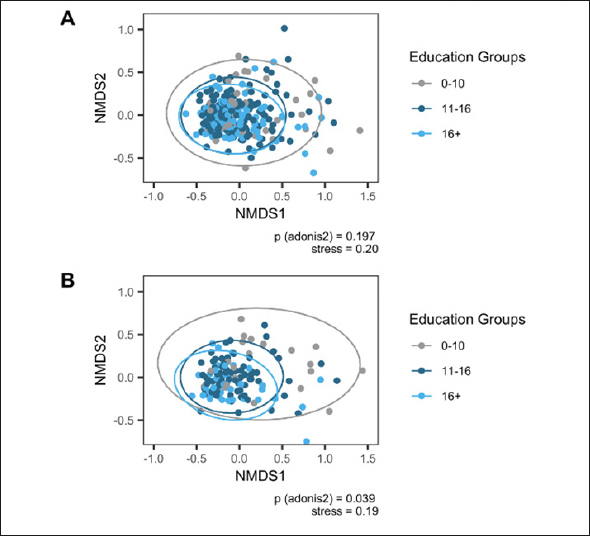
Ordination Plots for Education Groups Note. Ordination using Non-metric Multidimensional Scaling based on Bray-Curtis dissimilarity. P value (adonis2) adjusted for sex/gender, age, ATB, BDI-I, first language, PS, and APOE. A Full sample. B Restricted sample age 65 and older. Authors MK and VTEA.

Beta diversity differed significantly across education groups (betadisper: p=.048; adonis2: p=.04; Figure 2), when restricting to age 65 and older.

There were no significant differences in beta diversity between MCI or age groups (Supplementary Figure 2). As Chao1 likely reflects an underestimate of richness with ASVs based on DADA2, analyses were repeated with observed richness as measure of alpha diversity. These analyses yielded analogous findings (results not shown).

### Differential Abundance Analysis

DAA suggested higher relative abundance of Bacilli (class), Actinobacteria (class), Lactobacillales (order), Streptococcaceae (family), *Streptococcus* (genus), with DESeq 2 and *Lachnospiraceae* UCG 001 (genus) and two ASVs with ancombc in higher compared to lower (0–10 YEDU) education, adjusting for age, sex/gender, BMI, and ATB (Table 2).

**Table 2:** Taxonomic Analysis Across Groups of Education

		**DESeq 2**		**ancombc**	
**Level**	**Taxon**	**11–16**	**16+**	**11–16**	**16+**
Class	Bacilli†‡§		**		
	Actinobacteria†		*		
Order	Lactobacillales†‡§	**			
Family	Streptococcaceae†‡§	*			
Genus	Streptococcus†	*			
	Lachnospiraceae UCG 001†‡§				**
ASV	ASV 000053†				
	ASV 000508†				*

Note. * P Value < .05, ** P Value < .01, *** P Value < .001 (lowest P value of three adjustment sets). Orange indicates higher relative abundance in higher education compared with lower education (0–10 years). † Adjusted for age, sex/gender, BMI, and use of antibiotic medication in the last 6 months. ‡ Adjusted for age, sex/gender, BMI, use of antibiotic medication in the last 6 months, BDI-I, first language, and partnership status. § Adjusted for age, sex/gender, BMI, use of antibiotic medication in the last 6 months, BDI-I, first language, partnership status and APOE.

Sensitivity analysis with additional adjustments for BDI-I, first language, PS, and/or APOE replicated findings, except for Actinobacteria and *Streptococcus* with DESeq 2, and the two ASVs with ancombc. There was no overlap between DESeq 2 and ancombc (of note, p_adj_=0.07 for *Lachnospiraceae* UCG 001 with DESeq 2). Visual inspection of relative abundance plots suggests dose-response relationships of increasing YEDU and increasing relative abundance *Lachnospiraceae* UCG 001 (Supplementary Figure 3).

### Mediation by Alpha Diversity

With 0–10 YEDU as reference, higher education was associated with higher Chao1 (11–16 years=0.42 [95% CI 0.07, 0.77]; 16+ years=0.38 [95% CI 0.00, 0.76]; Supplementary Table 1). With an interaction term for education and Chao1 in the outcome model, higher education was associated to lower likelihood of MCI (11–16 years=−1.24 [95% CI −2.12, −0.35]; 16+ years=−1.26 [95% CI −2.22, −0.30]) whereas greater Chao1 was not significantly associated with MCI (coefficient=−0.14, [95% CI −0.76, 0.44]). Interaction terms were not significant (11–16 YEDU:Chao1=−0.02 [95% CI −0.80, 0.79]; 16+ YEDU:Chao1=−0.20 [95% CI −1.00, 0.62]). With Chao1 as mediator, NDE (16+ YEDU) was 0.35 ([95% CI 0.15, 0.81], p=.02, Table 3) and NIE (16+ YEDU) was 0.89 ([95% CI 0.68, 1.14], p=.33) suggesting an association of education to lower MCI risk, not mediated by Chao1 (total effect=0.31 [95% CI 0.14, 0.72], p=.008; CDE=0.33 [95% CI 0.14, 0.78], p=.02). The proportion eliminated, PE=0.22 ([95% CI 0.00, 0.59], p=.049), suggests most of the association of education on MCI risk being due to a direct effect of education but also a significant amount due to interaction, mediation, or both.

**Table 3:** Mediation Analysis with Chao1 as Mediator

	**Comparing 0–10 to 11–16 Years of Education**				**Comparing 0–10 to 16+ Years of EducationC**			
	**With Interaction**		**Without Interaction**		**With Interaction**		**Without Interaction**	
	**Estimate [95% CI]**	**P Value**	**Estimate [95% CI]**	**P Value**	**Estimate [95% CI]**	**P Value**	**Estimate [95% CI]**	**P Value**
Rcde	0.33 [0.15 to 0.76]	.012*	0.34 [0.16 to 0.74]	.010*	0.33 [0.14 to 0.78]	.018*	0.33 [0.15 to 0.76]	.015*
Rpnde	0.33 [0.15 to 0.73]	.012*	0.34 [0.16 to 0.74]	.010*	0.35 [0.15 to 0.81]	.016*	0.34 [0.15 to 0.76]	.015*
Rtnde	0.33 [0.15 to 0.77]	.011*	0.34 [0.16 to 0.74]	.010*	0.33 [0.15 to 0.79]	.016*	0.34 [0.15 to 0.76]	.015*
Rpnie	0.95 [0.70 to 1.19]	.647	0.92 [0.77 to 1.07]	.284	0.95 [0.71 to 1.17]	.662	0.93 [0.78 to 1.06]	.316
Rtnie	0.94 [0.71 to 1.33]	.693	0.92 [0.76 to 1.07]	.284	0.89 [0.68 to 1.14]	.334	0.93 [0.77 to 1.07]	.316
Rte	0.31 [0.15 to 0.68]	.006**	0.32 [0.15 to 0.68]	.006**	0.31 [0.14 to 0.72]	.008**	0.31 [0.14 to 0.71]	.008**
Ercde	−0.53 [−0.74 to −0.17]	.012*	-	-	−0.53 [−0.76 to −0.14]	.018*	-	-
Erintref	−0.14 [−0.32 to 0.05]	.114	-	-	−0.11 [−0.28 to 0.10]	.218	-	-
Erintmed	0.03 [−0.23 to 0.31]	.792	-	-	0.01 [−0.25 to 0.25]	.966	-	-
Erpnie	−0.05 [−0.30 to 0.19]	.647	-	-	−0.05 [−0.29 to 0.17]	.662	-	-
Ercde(P)	0.77 [0.42 to 0.99]	.008**	-	-	0.78 [0.41 to 1.00]	.010*	-	-
Erintref(P)	0.20 [−0.09 to 0.56]	.115	-	-	0.17 [−0.20 to 0.49]	.216	-	-
Erintmed(P)	−0.04 [−0.52 to 0.41]	.796	-	-	−0.01 [−0.41 to 0.45]	.964	-	-
Erpnie(P)	0.07 [−0.32 to 0.53]	.651	-	-	0.07 [-0.29 to 0.52]	.668	-	-
pm	0.03 [−0.15 to 0.28]	.694	0.04 [−0.04 to 0.25]	.288	0.06 [−0.06 to 0.38]	.340	0.03 [−0.04 to 0.24]	.321
int	0.16 [−0.05 to 0.40]	.104	-	-	0.16 [−0.02 to 0.38]	.082	-	-
pe	0.23 [0.01 to 0.58]	.043*	-	-	0.22 [0.00 to 0.59]	.049*	-	-

Note. Results of mediation analyses. Standard errors were estimated with 5000 bootstraps. * P Value < .05, ** P Value < .01, *** P Value < .001. Rcde: controlled direct effect odds ratio (referring to CDE); Rpnde: pure natural direct effect odds ratio (referring to NDE); Rtnde: total natural direct effect odds ratio; Rpnie: pure natural indirect effect odds ratio; Rtnie: total natural indirect effect odds ratio (referring to NIE); Rte: total effect odds ratio; Ercde: excess relative risk due to controlled direct effect; Erintref: excess relative risk due to reference interaction; Erintmed: excess relative risk due to mediated interaction; Erpnie: excess relative risk due to pure natural indirect effect; Ercde(P): proportion Ercde; Erintref(P): proportion Erintref; Erintmed(P): proportion Erintmed; Erpnie(P): proportion Erpnie; pm: overall proportion mediated; int: overall proportion attributable to interaction; pe: overall proportion eliminated. Cells with – indicate n/a.

Given the moderately rare outcome (∼22.5% MCI), CDE, NDE and NIE reported on the odds-ratio scale may be overestimated. As a sensitivity analysis, estimation was repeated on the risk-ratio scale using a multinomial log-linear link for the outcome model, resulting in a similar pattern of findings but without significant PE (results not shown) (37).

Analyses without interaction terms led to similar result patterns in regression models (Supplementary Table 1). Comparison of 0–10 to 11–16 YEDU led to similar result patterns in effect decomposition (Table 3). BMI was hypothesized as a potential mediator of education and MCI, or microbiome diversity and MCI, and thus not included in the main analyses but considered for robustness checks. Inclusion of BMI led to attenuated associations of education with Chao1 in the mediator, and of Chao1 with MCI in the outcome model. This in turn led to attenuated NIE and a similar, but no longer significant estimate of PE (results not shown).

Analyses with Shannon or inverse Simpson as mediator suggested similar findings but no significant PE. Analyses with inverse Simpson as mediator suggested similar findings except for no significant association of education with alpha diversity in the mediator model and a significant proportion of the total effect of education due to (additive) interaction, when comparing 0–10 to 11–16 YEDU (regression models: Supplementary Tables 2, 3, effect decomposition: Supplementary Tables 4, 5).

### Mediation by Beta Diversity

Ldm suggested no significant mediation by individual taxa or by the composition of the microbiome (p=.99 for n=48,000 completed permutations with ldm.omni3). Likewise, permanovaFL suggested no significant mediation by the composition of the microbiome, on the relative abundance (Bray-Curtis dissimilarity, p=.70), or presence-absence scale (Jaccard dissimilarity, p=.35), or overall (p=.54 for n=600 completed permutations with permanovaFL.omni). Robustness checks (with BMI) yielded a similar pattern of findings (results not shown).

## Discussion

Higher education was associated with a lower risk of MCI, with most of this association not being due to mediation by the gut microbiome. Despite differences in taxonomic signatures and gut microbiome composition between education groups, our findings suggest no significant mediation of the association of education with MCI by measures of alpha diversity or individual taxa. However, effect decomposition indicated potential additive interaction between education and alpha diversity.

### General Discussion

In this study, MCI risk was highest in the group with 0–10 YEDU. Higher education groups did not differ in their association with MCI. This reflects earlier findings suggesting that education is related to reserve capacity, and thus lower MCI risk, by in particular increasing levels of cognitive skills in early life which then persist until old age (4).

Critically, more than 16 YEDU likely reflect education beyond the end of adolescence, with positive effects levelling off and thus, no linear association of education with MCI.

Further analyses suggested a dominating direct effect of education. While education was associated with microbial diversity, no indicator of diversity was significantly associated to MCI, although less clear so for Chao1, reflecting richness, in models without interaction terms. Nonetheless, one fifth of the association of education on MCI could be removed (proportion eliminated) by intervening to fix Chao1 at the sample mean. Four-way decomposition suggests this to be most likely attributable to an additive interaction of education and Chao1, such that their association with lower MCI risk increases with increments in education (38). Of note, this finding reinforces most of the association of education with MCI to be flowing through a direct causal path, which is also supported by sensitivity analysis on the risk-ratio scale.

A potential explanation for the absence of statistically significant mediation would be that lower education may proxy higher MCI risk due to factors which are not associated with the gut microbiome, such as cognitive stimulation. In that case, the observed variation in gut microbiome diversity and composition across education groups would not be causally related to MCI risk.

However, our findings highlight education-related gut microbiome diversity and composition reflecting those found in MCI and AD. Given MCI as a strong risk factor and AD as the most common cause of dementia, similarities in the gut microbiota of individuals with low education – who are at higher risk of dementia – and of people living with AD may indicate further mechanisms contributing to the disease. These may involve nutritional choices and chronic low-grade inflammation or the synthesis of metabolites leading to modulation of nerve signaling via the enteric nervous system. A previous study found reduced richness as well as a distinct composition of the gut microbiome in terms of beta diversity in participants with AD compared to healthy controls (44). In line with a hypothesized neurodegenerative pathway involving education and the gut-brain-axis, our findings suggest that lower education is associated with reduced richness and a distinct gut microbiome composition. Conversely, another study found increasing richness with AD progression, which may be explained by an apparent gradient of education from lowest (in unimpaired cognition) to highest (in moderate AD) (45). Considering our findings lower education may not only have altered the likelihood of belonging to patient or control groups but may also have resulted in different taxonomic signatures.

Previous findings suggest similar alterations with respect to reduced alpha diversity in lower income and area-level SES settings (8, 9). Education is related to income and wealth, and consequently with selection into areas with fewer socioeconomic resources. As such, education may capture community-level or spatial exposures affecting gut microbiome composition (9).

Critically, we found Chao1 to be lower in older age, but only in participants with lower education. Additionally, compositional differences by education were only significant in older age. This extends on earlier reports of interindividual variability and reduced biodiversity in later life by suggesting education as a key modifier (46). Moreover, our finding of lower alpha diversity in lower education, suggests a putative association with a dysbiotic state. While lower alpha diversity has been discussed previously as a possible indicator of AD, to date, no concrete link has been established between education and dysbiosis and consequently AD or MCI (16, 47). This may be due to the relatively limited depth and linked resolution of sequencing or the breadth of education measures (16).

Extending on mediation results with alpha diversity metrics, ldm and permanovaFL did not identify mediating taxa or compositional changes translating into decreased MCI risk (40–42). However, DAA results suggest differential abundance in line with an MCI or AD phenotype and consequentially a potential communality of lower education and AD pathology. Bacilli (class), Actinobacteria (class), Lactobacillales (order), Streptococcaceae (family), *Streptococcus* (genus), *Lachnospiraceae* UCG 001 (genus) and two ASVs were depleted in lower education.

Contrary to our findings given a hypothesized link of education to MCI via the gut, previous studies showed an increased ratio of Firmicutes to Bacteroidetes (F/B), and an increased relative abundance of Lactobacillales in AD, and of Firmicutes in MCI (17, 48). However, earlier findings were likely driven by depleted Bacteroides; increases in Firmicutes were not statistically significant (17, 48). Other studies found increased Bacteroidetes in MCI without AD and, in line with our findings, depletion of Firmicutes in AD and amnestic MCI (7, 44, 49). Increased Bacteroides may relate to impaired cognition potentially through cerebral small vessel disease and resulting white matter hyperintensities and, in line with our findings, education may alter brain reserve to such damage via increased node degree (7, 50).

*Lachnospiraceae* UCG 001 were earlier found to be depleted in participants with more severe depressive symptoms, implying impaired synthesis of short-chain fatty-acids, such as butyrate and other depression-related neurotransmitters (51). Recent findings further suggest lower cognitive performance due to decreased levels of butyrate following stool transplantation of sleep deprived to control mice (52). Depletion of *Lachnospiraceae* UCG 001 in lower education may be associated to lower cognitive performance and MCI classification in line with a phenotype related to depressive symptom severity. Since we adjusted for BDI-I, education and depressive symptom severity may share a common neuroendocrinal pathway to impaired cognition, i.e., via nutritional choices, involving *Lachnospiraceae* UCG 001 and metabolites synthesized by gut microbiota.

Moreover, two differentially abundant ASV were identified, one classified as *Lachnospiraceae* UCG 001, the other as NK4A214 group, albeit classification comes with some uncertainty (Species unidentified, Supplementary Table 6).

One study found Actinobacteria and *Streptococcus* enriched in mild and moderate AD, potentially explained by higher educational attainment in AD-groups compared to controls, given our findings of higher abundance of Actinobacteria and *Streptococcus* in higher education (45). Consequently, both depleted Actinobacteria and *Streptococcus* in lower education may reflect an AD phenotype but the alternative explanation that their alteration reflects educational differences cannot be ruled out. Of note, Actinobacteria was earlier found depleted in AD compared to healthy controls suggesting e.g., detriments to intestinal barrier integrity in both AD and lower education (44).

### Limitations and Future Directions

In this study, we extensively triangulated potential mediation in a large cohort and point to potential interaction of education and gut microbiome diversity regarding MCI risk. Despite careful adjustment, residual confounding may bias results. Effect decomposition assumes no unmeasured (i) exposure-outcome, (ii) mediator-outcome, or (iii) exposure-mediator confounding, and (iv) that (ii)-confounders are not affected by the exposure. However, PE and CDE do not require (iii) or (iv), and NDE is robust to (iv), assuming monotone associations (53, 54). MCI classification was based on a screening instrument. Differences in causes underlying MCI classification may bias DAA, which we could not formally assess (3). Moreover, SES was not formally addressed in the present analyses and may reflect a common cause of or indirect causal path variable of educational differences and MCI risk. However, DAA was carefully adjusted for different confounder sets, including lifestyle-related variables and risk factors of impaired cognition, such as BMI, depressive symptoms, or partnership status. Grouping of education may bias estimates, although the associations of YEDU with MCI is likely non-linear. Further, compulsory schooling years vary across birthyears (29). Grouping by less than 10 YEDU selects older participants or those that immigrated. However, analysis was adjusted for age and first language as proxy for immigration. Limited diversity and sample size in this cohort prevented subgroup analysis and hampers generalizability (Supplementary Table 7). Further analysis regarding functional categories and diversity are necessary to fully elucidate implications of distinct taxonomic signatures (55).

## Conclusion

Our results suggest signatures of the gut microbiome that have been identified previously in AD and MCI to be reflected in lower education. We show that most of the association of education with MCI is of a direct nature and stress the importance of considering social determinants of health, specifically education, as key modifiers in microbiome studies. Our findings underline the potential of the gut microbiome as a biomarker and intervention target regarding MCI, which is promising, considering its modifiability until later life. Future research with longitudinal survey designs is required to further investigate potential interaction of education and the gut microbiome and their implication for neurodegenerative diseases.

### Supplementary Material

Supplementary material, approximately 1.50 MB.

## Notes

*Ethical standards:* The Luxembourg Parkinson’s Study (LUXPARK) of the National Centre of Excellence in Research on Parkinson’s disease (NCER-PD) received approval from the National Ethics Board (CNER Ref: 201407/13) and Data Protection Committee (CNPD Ref: 446/2017) and was conducted according to the Declaration of Helsinki. All participants provided written informed consent.
