# Supplementary material for: Education as Risk Factor of Mild Cognitive Impairment: The Link to the Gut Microbiome
Source: J Prev Alzheimers Dis. 2024 Jan 23;11(3):759–68. doi: 10.14283/jpad.2024.19 (PMC11060993; doi:10.14283/jpad.2024.19)
Supplement: Supplementary file 1 — Supplementary material, approximately 1.50 MB. [file 42414_2024_305_MOESM1_ESM.docx]

# Supplementary Material

# Diversity Measures

**Alpha Diversity:**

Chao1 was computed as an indicator of richness, which ignores abundance and increases with the number of prevalent species. [1] The Shannon and inverse Simpson indices were computed as indicators of both richness and evenness. They increase with the number of prevalent species and penalize presence of microbes dominating in prevalence. [2] Measures were computed after rarefication (*estimate_richness* [*phyloseq*]; *rarefy_even_depth* [*phyloseq*]) [3]

**Beta Diversity:**

Percentage difference, referred to sometimes as Bray-Curtis dissimilarity, and Jaccard distance were computed, which reflect the fraction of the number of unique species between individuals and the number of shared, and unique species (*vegdist* [*vegan*]). [4–6]

# R Session Info (Excerpt)

─ Session info ───────────────────────────────────────────────────────────────────

version R version 4.3.1 (2023-06-16)

os macOS Ventura 13.5

system x86_64, darwin20

ui RStudio

language (EN)

rstudio 2023.06.2+561 Mountain Hydrangea (desktop)

─ Packages ───────────────────────────────────────────────────────────────────────

ANCOMBC * 2.2.1 2023-07-06 [1] Bioconductor

CMAverse * 0.1.0 2023-09-09 [1] Github (BS1125/CMAverse@fa8ccab)

DESeq2 * 1.40.2 2023-06-25 [1] Bioconductor

LDM * 6.0 2023-09-04 [1] CRAN (R 4.3.0)

phyloseq * 1.44.0 2023-05-11 [1] Bioconductor

vegan * 2.6-4 2022-10-11 [1] CRAN (R 4.3.0)

# Differential Abundance Analysis

Since abundance reflects count data that is compositional (i.e. abundance of one species directly affects abundance of others), overdispersed and skewed (i.e. high variance and frequent zero counts for rare species), traditional approaches to statistical inference may lead to inflated false discovery rates and are thus not applicable. [7,8] Multiple methods have been developed to overcome these caveats, but they may provide discordant results in complex disease settings. Here applied approaches were the analysis of compositions of microbiomes with bias correction (*ancombc* [*ANCOMBC*]) and moderated estimation of fold change and dispersion (*DESeq* [*DESeq2*]). *DESeq* applies negative binomial generalized linear models and *ancombc* implements bias correction inherent to the underlying abundance quantification. For a more detailed description see function descriptions in packages *DESeq2* and *ANCOMBC.* [8–11]

# R Code Mediation Analysis with CMAverse (Example with Chao1)

do_cmest = function(data,

mediator,

a, astar = "0-10",

mval,

yreg = "logistic",

int = FALSE,

print = F){

set.seed(123)

med_analysis_1 <- cmest(data = data,

model = "rb",

outcome = "MCI_bin",

exposure = "Years_of_Education",

mediator = c(mediator),

basec = c("Age",

"Gender",

"ATB_in_last_6_months",

#"BMI",

"BDI_I_mild",

"First_Language",

"Living_With_Partner",

"APOE4"),

mreg = list("linear"),

yreg = yreg,

EMint = int,

astar = astar,

a = a,

mval = mval,

yval = 1,

estimation = "imputation",

inference = "bootstrap",

nboot = 5000)

if(print) {med_analysis_1 %>% summary() %>% print()}

return(med_analysis_1)

}

# function call example

clin_df = ncer_adiv %>%

mutate(Chao1 = as.vector(scale(Chao1)))

res16_Chao1_int = do_cmest(clin_df,

mediator = "Chao1",

a = "16+",

mval = list(0),

int = T)

# R Code Mediation Analysis with *Ldm* and *PermanovaFL*

# required formula

# otu.table | (set of confounders) ~ (set of exposures) + (set of outcomes)

sample_tab = ncer_phyloseqs$Genus %>% sample_data %>% as_tibble %>% as.data.frame

otu_tab = ncer_phyloseqs$Genus %>% otu_table() %>% t() %>% as.data.frame()

# ldm med

res.ldm.med <- ldm(formula = otu_tab |

(Age + Gender + ATB_in_last_6_months + BDI_I_mild + First_Language + Living_With_Partner + APOE4) ~

(Years_of_Education) + # exposure

(MCI), # outcome

data=clin_df,

seed=67817,

n.cores=12,

test.mediation=TRUE,

test.omni3 = T)

# permanova med

res.perm.med = permanovaFL(otu_tab |

(Age + Gender + ATB_in_last_6_months + BDI_I_mild + First_Language + Living_With_Partner + APOE4) ~

(Years_of_Education) + # exposure

(MCI), # outcome

data=clin_df, seed=82955,

test.mediation = T,

dist.method = c("jaccard", "bray"),

binary = c(TRUE, FALSE))

Note that *ldm* and *permanovaFL* preclude effect decomposition. While other approaches to mediation analysis with multiple mediators and effect decomposition exist, the assumption of multivariate normal distribution among mediators is not met for compositional abundance data. [7] Further, given the large number of mediators, close to sample size, regularization would be required. [12,13]

# NDE and NIE

We acknowledge that temporality, necessary for causal interpretation, is not given with cross-sectional data. However, the cited literature refers to the term ‘effects’ hence this statistical terminology is used to avoid misinterpretation. Here, NDE refers to the difference in the counterfactual outcome, fixing the mediator to the level it would have taken with education 0-10 years, and intervening to change education from 0-10 years to >10 years (i.e. pure natural direct effect) [14]. Hence, NDE describes the effect of education on MCI irrespective of alpha diversity. Note that CDE describes the effect of education on MCI for a specified level of alpha diversity, i.e., the sample mean. The NIE refers to the difference in the counterfactual outcome, fixing education to >10 years and intervening to change the mediator from the level it would have taken in 0-10 years to the level it would have taken in >10 years (i.e. total indirect effect). [14] Hence, NIE describes the effect of education on MCI only passing through alpha diversity. [15] Note that NDE and NIE reflect direct and indirect effects obtained using the approach of Baron and Kenny in absence of interaction between education and microbiome diversity. [16,17]

1. $E\left[ Y_{a, M(a^{*})}-Y_{a^{*}, M(a^{*})}|C=c \right]$
2. $E\left[ Y_{a, M\left( a \right)}-Y_{a, M\left( a^{*} \right)}|C=c \right]$

With

exposure a* at reference level and exposure a at intervention level,
mediator M observed at a* or a, conditional on covariates C = c

# Table 1. Coefficients of Regression Models with Chao1.

|  | **Mediator Model** | | | **Outcome Model** | | | | | |
| --- | --- | --- | --- | --- | --- | --- | --- | --- | --- |
|  |  |  |  | **With Interaction** | | | **Without Interaction** | | |
|  | **Estimate** **[95% CI]** | ***P*** |  | **Estimate** **[95% CI]** | ***P*** |  | **Estimate** **[95% CI]** | ***P*** |  |
| **YEDU** |  |  |  |  |  |  |  |  |  |
| 0-10 |  |  |  | [Reference] |  |  |  |  |  |
| 11-16 | 0.42 [ 0.07 to 0.77] | .018 | * | -1.24 [-2.12 to -0.35] | .005 | ** | -1.21 [-2.07 to -0.35] | .006 | ** |
| 16+ | 0.38 [ 0.00 to 0.76] | .050 |  | -1.26 [-2.22 to -0.30] | .010 | * | -1.23 [-2.16 to -0.31] | .009 | ** |
| **Chao1** | - | - |  | -0.14 [-0.76 to 0.44] | .640 |  | -0.21 [-0.54 to 0.11] | .198 |  |
| **YEDU:Chao1** | - | - |  |  |  |  | - | - |  |
| **11-16:Chao1** | - | - |  | -0.02 [-0.80 to 0.79] | .967 |  | - | - |  |
| **16+:Chao1** | - | - |  | -0.20 [-1.00 to 0.62] | .630 |  | - | - |  |

*Note.* Regression coefficients for mediator and outcome models, used for mediation analysis with alpha diversity measures. YEDU = Years of education. All analyses were adjusted for age, sex/gender, ATB, BDI-I, first language, PS and *APOE*. * *P* Value < .05, ** *P* Value < .01, *** *P* Value < .001.

# Table 2. Coefficients of Regression Models with Shannon.

|  | **Mediator Model** | | | | | **Outcome Model** | | | | | |
| --- | --- | --- | --- | --- | --- | --- | --- | --- | --- | --- | --- |
|  |  |  | |  | | **With Interaction** | | | **Without Interaction** | | |
|  | **Estimate** **[95% CI]** | | ***P* Value** | |  | **Estimate** **[95% CI]** | ***P* Value** |  | **Estimate** **[95% CI]** | ***P* Value** |  |
| **Years of Education** |  | |  | |  |  |  |  |  |  |  |
| 0-10 |  | |  | |  | [Reference] |  |  |  |  |  |
| 11-16 | 0.36 [ 0.01 to 0.71] | | .042 | | * | -1.31 [-2.18 to -0.45] | .003 | ** | -1.26 [-2.11 to -0.41] | .003 | ** |
| 16+ | 0.37 [-0.01 to 0.75] | | .055 | |  | -1.28 [-2.22 to -0.36] | .007 | ** | -1.25 [-2.18 to -0.34] | .007 | ** |
| **Shannon** | - | | - | |  | -0.01 [-0.61 to 0.59] | .985 |  | -0.13 [-0.46 to 0.19] | .423 |  |
| **Years of Education:Shannon** | - | | - | |  |  |  |  | - | - |  |
| **11-16:Shannon** | - | | - | |  | 0.04 [-0.77 to 0.87] | .924 |  | - | - |  |
| **16+:Shannon** | - | | - | |  | -0.38 [-1.19 to 0.43] | .354 |  | - | - |  |

*Note.* Regression coefficients for mediator and outcome models, used for mediation analysis with alpha diversity measures. All analyses were adjusted for age, sex/gender, ATB, BDI-I, first language, PS and *APOE*. * *P* Value < .05, ** *P* Value < .01, *** *P* Value < .001.

# Table 3. Coefficients of Regression Models with Inverse Simpson.

|  | **Mediator Model** | | | | | **Outcome Model** | | | | | |
| --- | --- | --- | --- | --- | --- | --- | --- | --- | --- | --- | --- |
|  |  |  | |  | | **With Interaction** | | | **Without Interaction** | | |
|  | **Estimate** **[95% CI]** | | ***P* Value** | |  | **Estimate** **[95% CI]** | ***P* Value** |  | **Estimate** **[95% CI]** | ***P* Value** |  |
| **Years of Education** |  | |  | |  |  |  |  |  |  |  |
| 0-10 |  | |  | |  | [Reference] |  |  |  |  |  |
| 11-16 | 0.17 [-0.18 to 0.52] | | .342 | |  | -1.33 [-2.18 to -0.48] | .002 | ** | -1.29 [-2.14 to -0.46] | .002 | ** |
| 16+ | 0.22 [-0.16 to 0.61] | | .260 | |  | -1.30 [-2.23 to -0.39] | .005 | ** | -1.28 [-2.21 to -0.38] | .006 | ** |
| **Inverse Simpson** | - | | - | |  | -0.01 [-0.68 to 0.63] | .975 |  | -0.08 [-0.42 to 0.25] | .625 |  |
| **Years of Education:Inverse Simpson** | - | | - | |  |  |  |  | - | - |  |
| **11-16:Inverse Simpson** | - | | - | |  | 0.19 [-0.62 to 1.02] | .649 |  | - | - |  |
| **16+:Inverse Simpson** | - | | - | |  | -0.50 [-1.41 to 0.41] | .282 |  | - | - |  |

*Note.* Regression coefficients for mediator and outcome models, used for mediation analysis with alpha diversity measures. All analyses were adjusted for age, sex/gender, ATB, BDI-I, first language, PS and *APOE*. * *P* Value < .05, ** *P* Value < .01, *** *P* Value < .001.

# Table 4. Mediation Analysis with Shannon Index as Mediator.

| **Comparing 0-10 to 11-16 Years of Education** | | | | | | | | **Comparing 0-10 to 16+ Years of Education** | | | | | |
| --- | --- | --- | --- | --- | --- | --- | --- | --- | --- | --- | --- | --- | --- |
|  | **With Interaction** |  |  | **Without Interaction** | |  | **With Interaction** | |  |  | **Without Interaction** | |  |
|  | **Estimate [95% CI]** | ***P*** |  | **Estimate [95% CI]** | ***P*** |  | **Estimate [95% CI]** | | ***P*** |  | **Estimate [95% CI]** | ***P*** |  |
| **Rcde** | 0.31 [ 0.14 to 0.69] | .007 | ** | 0.32 [ 0.15 to 0.69] | .007 | ** | 0.32 [ 0.13 to 0.74] | | .012 | * | 0.33 [ 0.14 to 0.75] | .014 | * |
| **Rpnde** | 0.31 [ 0.14 to 0.69] | .007 | ** | 0.32 [ 0.15 to 0.70] | .007 | ** | 0.36 [ 0.15 to 0.86] | | .022 | * | 0.33 [ 0.14 to 0.75] | .014 | * |
| **Rtnde** | 0.31 [ 0.14 to 0.70] | .008 | ** | 0.32 [ 0.15 to 0.70] | .007 | ** | 0.32 [ 0.14 to 0.74] | | .011 | * | 0.33 [ 0.14 to 0.75] | .014 | * |
| **Rpnie** | 1.00 [ 0.80 to 1.25] | .986 |  | 0.96 [ 0.83 to 1.09] | .526 |  | 1.00 [ 0.79 to 1.26] | | .985 |  | 0.96 [ 0.81 to 1.10] | .531 |  |
| **Rtnie** | 1.01 [ 0.79 to 1.40] | .897 |  | 0.96 [ 0.82 to 1.10] | .526 |  | 0.88 [ 0.65 to 1.09] | | .260 |  | 0.96 [ 0.81 to 1.10] | .531 |  |
| **Rte** | 0.31 [ 0.15 to 0.67] | .005 | ** | 0.31 [ 0.15 to 0.67] | .004 | ** | 0.32 [ 0.15 to 0.72] | | .009 | ** | 0.31 [ 0.14 to 0.71] | .008 | ** |
| **Ercde** | -0.56 [-0.77 to -0.22] | .007 | ** | - |  | - | -0.55 [-0.78 to -0.18] | | .012 | * | - | - |  |
| **Erintref** | -0.13 [-0.26 to 0.03] | .093 |  | - | - |  | -0.09 [-0.21 to 0.17] | | .344 |  | - | - |  |
| **Erintmed** | 0.01 [-0.26 to 0.23] | .961 |  | - | - |  | -0.04 [-0.37 to 0.17] | | .677 |  | - | - |  |
| **Erpnie** | 0.00 [-0.20 to 0.25] | .986 |  | - | - |  | 0.00 [-0.21 to 0.26] | | .985 |  | - | - |  |
| **Ercde(P)** | 0.81 [ 0.58 to 1.06] | .003 | ** | - | - |  | 0.80 [ 0.56 to 1.05] | | .005 | ** | - | - |  |
| **Erintref(P)** | 0.19 [-0.07 to 0.46] | .094 |  | - | - |  | 0.13 [-0.34 to 0.34] | | .336 |  | - | - |  |
| **Erintmed(P)** | -0.01 [-0.43 to 0.44] | .963 |  | - | - |  | 0.06 [-0.28 to 0.71] | | .682 |  | - | - |  |
| **Erpnie(P)** | 0.00 [-0.42 to 0.35] | .988 |  | - | - |  | 0.00 [-0.43 to 0.37] | | .988 |  | - | - |  |
| **pm** | -0.01 [-0.21 to 0.17] | .898 |  | 0.02 [-0.06 to 0.17] | .527 |  | 0.06 [-0.05 to 0.49] | | .266 |  | 0.02 [-0.05 to 0.20] | .535 |  |
| **int** | 0.19 [-0.03 to 0.45] | .070 |  | - | - |  | 0.19 [ 0.00 to 0.49] | | .053 |  | - | - |  |
| **pe** | 0.19 [-0.06 to 0.42] | .107 |  | - | - |  | 0.20 [-0.05 to 0.44] | | .089 |  | - | - |  |

*Note.* Results of mediation analysis with interaction terms of education and Chao1 in the outcome model. Standard errors were estimated with 2000 bootstraps. * *P* Value < .05, ** *P* Value < .01, *** *P* Value < .001. Rcde: controlled direct effect odds ratio (referring to CDE); Rpnde: pure natural direct effect odds ratio (referring to NDE); Rtnde: total natural direct effect odds ratio; Rpnie: pure natural indirect effect odds ratio; Rtnie: total natural indirect effect odds ratio (referring to NIE); Rte: total effect odds ratio; Ercde: excess relative risk due to controlled direct effect; Erintref: excess relative risk due to reference interaction; Erintmed: excess relative risk due to mediated interaction; Erpnie: excess relative risk due to pure natural indirect effect; Ercde(P): proportion Ercde; Erintref(P): proportion Erintref; Erintmed(P): proportion Erintmed; Erpnie(P): proportion Erpnie; pm: overall proportion mediated; int: overall proportion attributable to interaction; pe: overall proportion eliminated). Cells with – indicate n/a.

# Table 5. Mediation Analysis with Inverse Simpson Index as Mediator.

| **Comparing 0-10 to 11-16 Years of Education** | | | | | | | | **Comparing 0-10 to 16+ Years of Education** | | | | | |
| --- | --- | --- | --- | --- | --- | --- | --- | --- | --- | --- | --- | --- | --- |
|  | **With Interaction** |  |  | **Without Interaction** | |  | **With Interaction** | |  |  | **Without Interaction** | |  |
|  | **Estimate [95% CI]** | ***P*** |  | **Estimate [95% CI]** | ***P*** |  | **Estimate [95% CI]** | | ***P*** |  | **Estimate [95% CI]** | ***P*** |  |
| **Rcde** | 0.31 [ 0.14 to 0.68] | .005 | ** | 0.31 [ 0.15 to 0.67] | .005 | ** | 0.32 [ 0.13 to 0.73] | | .010 | * | 0.32 [ 0.14 to 0.73] | .009 | ** |
| **Rpnde** | 0.30 [ 0.15 to 0.66] | .004 | ** | 0.31 [ 0.15 to 0.67] | .005 | ** | 0.36 [ 0.16 to 0.83] | | .020 | * | 0.32 [ 0.14 to 0.73] | .009 | ** |
| **Rtnde** | 0.31 [ 0.15 to 0.69] | .006 | ** | 0.31 [ 0.15 to 0.67] | .005 | ** | 0.32 [ 0.14 to 0.75] | | .010 | * | 0.32 [ 0.14 to 0.73] | .009 | ** |
| **Rpnie** | 1.00 [ 0.85 to 1.20] | .964 |  | 0.99 [ 0.89 to 1.08] | .832 |  | 1.00 [ 0.83 to 1.25] | | .991 |  | 0.98 [ 0.87 to 1.09] | .762 |  |
| **Rtnie** | 1.03 [ 0.89 to 1.24] | .705 |  | 0.99 [ 0.88 to 1.08] | .832 |  | 0.91 [ 0.67 to 1.10] | | .370 |  | 0.98 [ 0.86 to 1.10] | .762 |  |
| **Rte** | 0.31 [ 0.15 to 0.67] | .005 | ** | 0.31 [ 0.15 to 0.67] | .005 | ** | 0.32 [ 0.15 to 0.73] | | .009 | ** | 0.31 [ 0.14 to 0.71] | .008 | ** |
| **Ercde** | -0.56 [-0.77 to -0.24] | .005 | ** | - | - |  | -0.55 [-0.78 to -0.20] | | .010 | * | - | - |  |
| **Erintref** | -0.13 [-0.25 to 0.00] | .055 |  | - | - |  | -0.09 [-0.19 to 0.17] | | .326 |  | - | - |  |
| **Erintmed** | 0.01 [-0.19 to 0.18] | .966 |  | - | - |  | -0.03 [-0.36 to 0.12] | | .701 |  | - | - |  |
| **Erpnie** | 0.00 [-0.15 to 0.20] | .964 |  | - | - |  | 0.00 [-0.17 to 0.25] | | .991 |  | - | - |  |
| **Ercde(P)** | 0.82 [ 0.61 to 1.02] | .004 | ** | - | - |  | 0.81 [ 0.58 to 1.10] | | .003 | ** | - | - |  |
| **Erintref(P)** | 0.20 [ 0.00 to 0.44] | .054 |  | - | - |  | 0.14 [-0.35 to 0.33] | | .318 |  | - | - |  |
| **Erintmed(P)** | -0.01 [-0.33 to 0.33] | .965 |  | - | - |  | 0.05 [-0.21 to 0.67] | | .705 |  | - | - |  |
| **Erpnie(P)** | 0.00 [-0.34 to 0.27] | .965 |  | - | - |  | 0.00 [-0.40 to 0.31] | | .990 |  | - | - |  |
| **pm** | -0.01 [-0.14 to 0.07] | .705 |  | 0.01 [-0.05 to 0.09] | .833 |  | 0.05 [-0.07 to 0.41] | | .375 |  | 0.01 [-0.05 to 0.12] | .764 |  |
| **int** | 0.18 [ 0.02 to 0.40] | .039 | * | - | - |  | 0.18 [-0.04 to 0.48] | | .084 |  | - | - |  |
| **pe** | 0.18 [-0.02 to 0.39] | .066 |  | - | - |  | 0.19 [-0.10 to 0.42] | | .114 |  | - | - |  |

*Note.* Results of mediation analysis with interaction terms of education and Chao1 in the outcome model. Standard errors were estimated with 2000 bootstraps. * *P* Value < .05, ** *P* Value < .01, *** *P* Value < .001. Rcde: controlled direct effect odds ratio (referring to CDE); Rpnde: pure natural direct effect odds ratio (referring to NDE); Rtnde: total natural direct effect odds ratio; Rpnie: pure natural indirect effect odds ratio; Rtnie: total natural indirect effect odds ratio (referring to NIE); Rte: total effect odds ratio; Ercde: excess relative risk due to controlled direct effect; Erintref: excess relative risk due to reference interaction; Erintmed: excess relative risk due to mediated interaction; Erpnie: excess relative risk due to pure natural indirect effect; Ercde(P): proportion Ercde; Erintref(P): proportion Erintref; Erintmed(P): proportion Erintmed; Erpnie(P): proportion Erpnie; pm: overall proportion mediated; int: overall proportion attributable to interaction; pe: overall proportion eliminated). Cells with – indicate n/a.

# Table 6. Taxonomic Classification of Identified Taxa.

| **Taxon** | **Domain** | **Phylum** | **Class** | **Order** | **Family** | **Genus** |
| --- | --- | --- | --- | --- | --- | --- |
| **Bacilli** | Bacteria | Firmicutes | Bacilli | NA | NA | NA |
| **Actinobacteria** | Bacteria | Actinobacteriota | Actinobacteria | NA | NA | NA |
| **Lactobacillales** | Bacteria | Firmicutes | Bacilli | Lactobacillales | NA | NA |
| **Streptococcaceae** | Bacteria | Firmicutes | Bacilli | Lactobacillales | Streptococcaceae | NA |
| **Streptococcus** | Bacteria | Firmicutes | Bacilli | Lactobacillales | Streptococcaceae | Streptococcus |
| **Lachnospiraceae UCG 001** | Bacteria | Firmicutes | Clostridia | Lachnospirales | Lachnospiraceae | Lachnospiraceae UCG 001 |
| **ASV 000508** | Bacteria | Firmicutes | Clostridia | Lachnospirales | Lachnospiraceae | Lachnospiraceae UCG 001 |
| **ASV 000053** | Bacteria | Firmicutes | Clostridia | Oscillospirales | Oscillospiraceae | NK4A214_group |

*Note.* Taxonomic classification as identified with *DESeq2* and *ancombc*.

# Table 7. First Languages Spoken.

| **First Language** | **NC (n=200)** | **MCI (n=58)** |
| --- | --- | --- |
| **Danish** | 3 | 0 |
| **Dutch** | 2 | 1 |
| **English** | 7 | 0 |
| **French** | 27 | 5 |
| **German** | 20 | 3 |
| **Hungarian** | 1 | 0 |
| **Italian** | 2 | 2 |
| **Luxembourgish** | 135 | 44 |
| **Portuguese** | 1 | 3 |
| **Slovene** | 1 | 0 |
| **Spanish** | 1 | 0 |

# Figure 1. Alpha Diversity Across Education Groups.


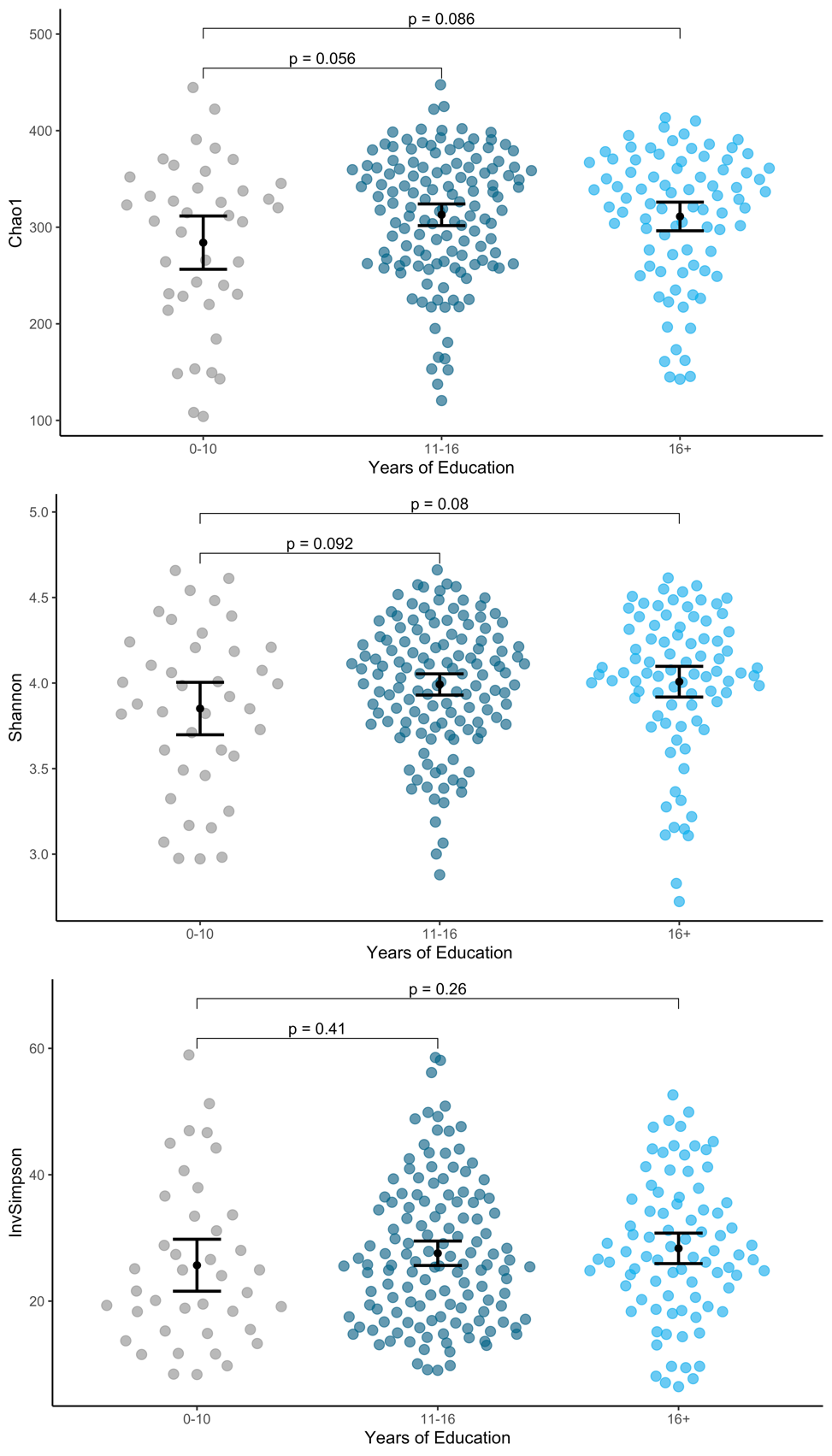


*Note.* Panels show results stratified by education groups with 0-10, 11-16 and 16+ years of education. Reported *P* values refer to Student’s t-Tests. InvSimpson = Inverse Simpson. Author MK.

# Figure 2. Ordination Plots for MCI and Age Groups.

**A MCI Groups**


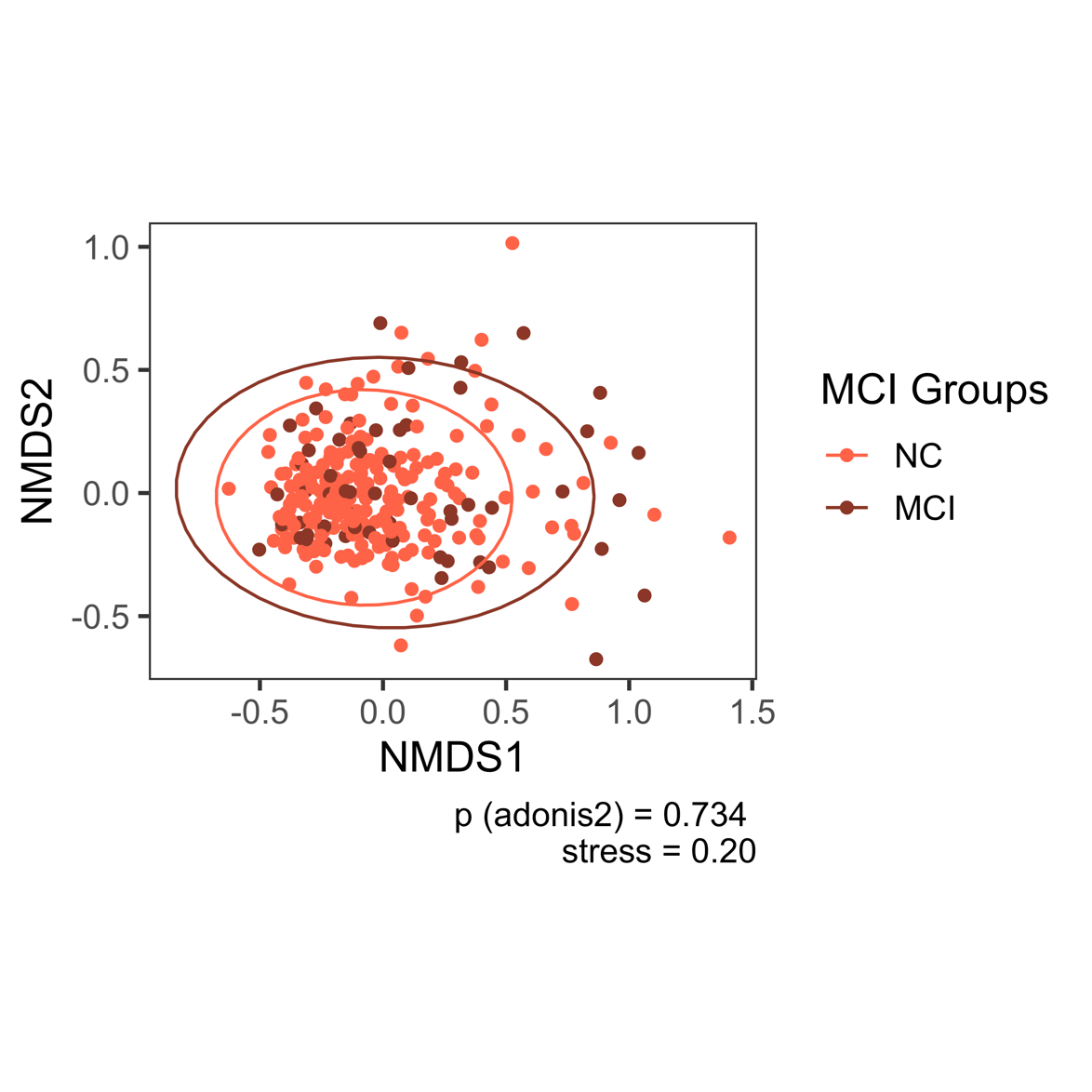


Ordination using Non-metric Multidimensional Scaling based on Bray-Curtis dissimilarity. *P* value (*adonis2*) adjusted for MCI, education, gender, age, ATB, BDI-I, first language, PS, *APOE*. Authors MK and VTEA.

**B Age Groups**


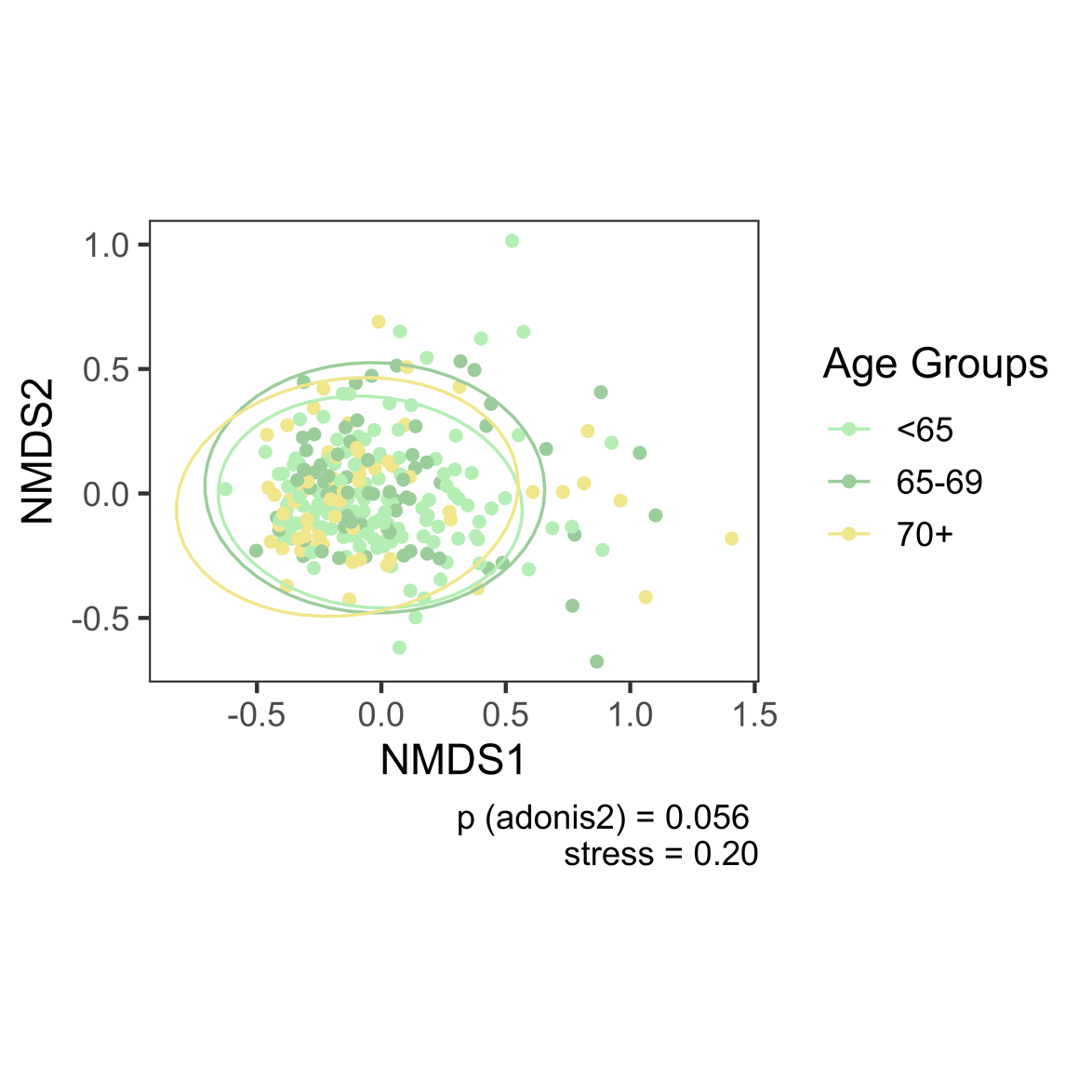


Ordination using Non-metric Multidimensional Scaling based on Bray-Curtis dissimilarity. *P* value (*adonis2*) adjusted for age categories, education, gender, ATB, BDI-I, first language, PS, *APOE*. Authors MK and VTEA.

# Figure 3. Relative Abundance Across Groups of Education.


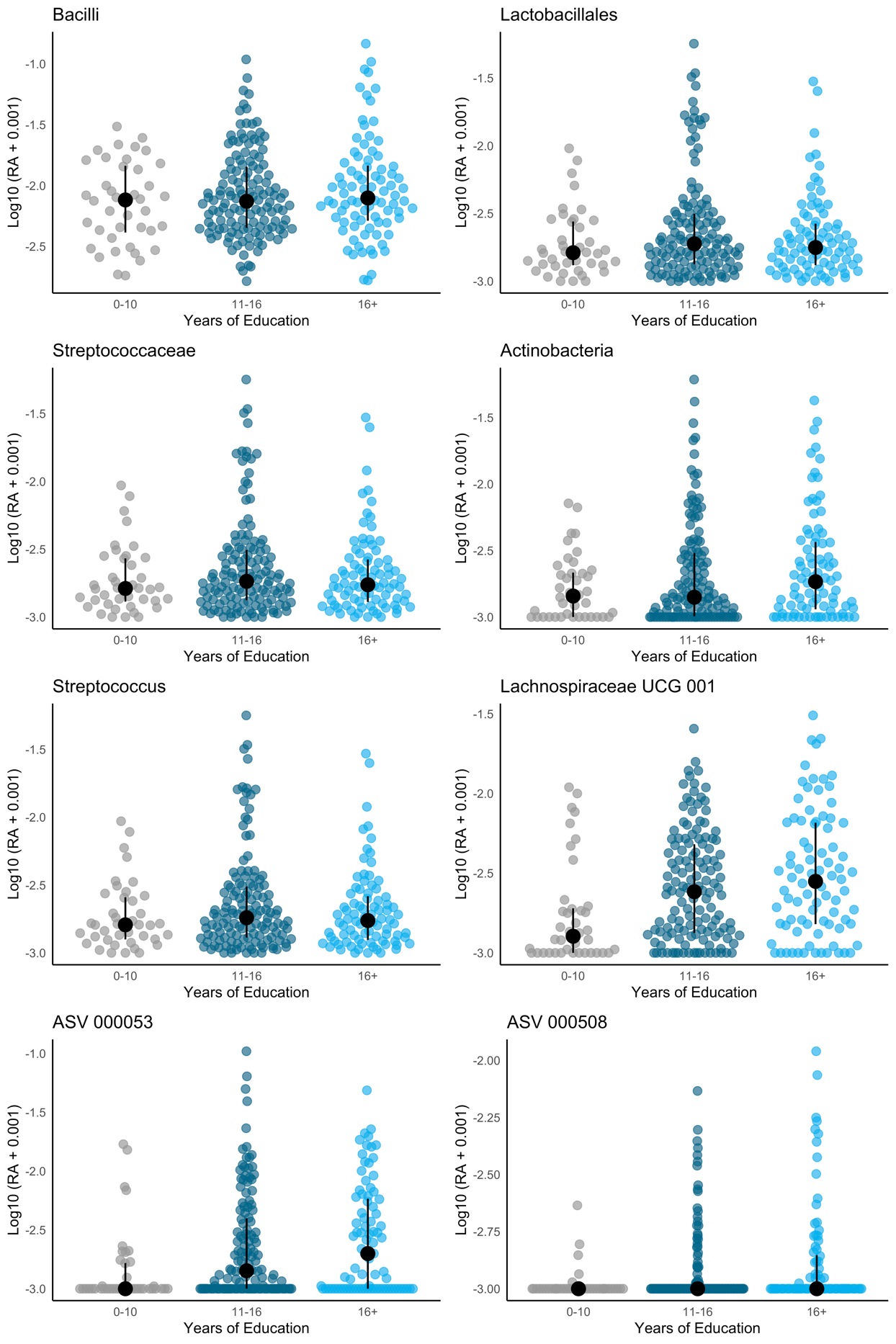


*Note*. Y axis shows log10-transformed relative abundance plus 10^-3^. Bars indicate median and interquartile range. Authors MK and VTEA.

# References

[1] Chao A. Nonparametric Estimation of the Number of Classes in a Population. Scand J Stat 1984;11:265–70. https://www.jstor.org/stable/4615964.

[2] Hill TCJ, Walsh KA, Harris JA, Moffett BF. Using ecological diversity measures with bacterial communities. FEMS Microbiol Ecol 2003;43:1–11. https://doi.org/10.1111/j.1574-6941.2003.tb01040.x.

[3] McMurdie PJ, Holmes S. phyloseq: An R Package for Reproducible Interactive Analysis and Graphics of Microbiome Census Data. PLOS ONE 2013;8:e61217. https://doi.org/10.1371/journal.pone.0061217.

[4] Faith DP, Minchin PR, Belbin L. Compositional dissimilarity as a robust measure of ecological distance. Vegetatio 1987;69:57–68. https://doi.org/10.1007/BF00038687.

[5] Legendre P, De Cáceres M. Beta diversity as the variance of community data: dissimilarity coefficients and partitioning. Ecol Lett 2013;16:951–63. https://doi.org/10.1111/ele.12141.

[6] Oksanen J., Simpson G. L., Blanchet F. G., Kindt R., Legendre P., Minchin P., et al. vegan: Community Ecology Package. R package version 2.6-2, 2022.

[7] Aitchison J. The Statistical Analysis of Compositional Data. J R Stat Soc Ser B Methodol 1982;44:139–60. https://doi.org/10.1111/j.2517-6161.1982.tb01195.x.

[8] Mandal S, Van Treuren W, White RA, Eggesbø M, Knight R, Peddada SD. Analysis of composition of microbiomes: a novel method for studying microbial composition. Microb Ecol Health Dis 2015;26:27663. https://doi.org/10.3402/mehd.v26.27663.

[9] Love MI, Huber W, Anders S. Moderated estimation of fold change and dispersion for RNA-seq data with DESeq2. Genome Biol 2014;15:550. https://doi.org/10.1186/s13059-014-0550-8.

[10] Lin H, Peddada SD. Analysis of compositions of microbiomes with bias correction. Nat Commun 2020;11:3514. https://doi.org/10.1038/s41467-020-17041-7.

[11] Kaul A, Mandal S, Davidov O, Peddada SD. Analysis of Microbiome Data in the Presence of Excess Zeros. Front Microbiol 2017;8:2114. https://doi.org/10.3389/fmicb.2017.02114.

[12] VanderWeele TJ, Vansteelandt S. Mediation Analysis with Multiple Mediators. Epidemiol Methods 2014;2:95–115. https://doi.org/10.1515/em-2012-0010.

[13] Yue Y, Hu Y-J. Extension of PERMANOVA to Testing the Mediation Effect of the Microbiome. Genes 2022;13:940. https://doi.org/10.3390/genes13060940.

[14] VanderWeele TJ. A unification of mediation and interaction: a four-way decomposition. Epidemiol Camb Mass 2014;25:749. https://doi.org/10.1097/EDE.0000000000000121.

[15] Richiardi L, Bellocco R, Zugna D. Mediation analysis in epidemiology: methods, interpretation and bias. Int J Epidemiol 2013;42:1511–9. https://doi.org/10.1093/ije/dyt127.

[16] Valeri L, VanderWeele TJ. Mediation analysis allowing for exposure-mediator interactions and causal interpretation: theoretical assumptions and implementation with SAS and SPSS macros. Psychol Methods 2013;18:137–50. https://doi.org/10.1037/a0031034.

[17] Baron RM, Kenny DA. The moderator–mediator variable distinction in social psychological research: Conceptual, strategic, and statistical considerations. J Pers Soc Psychol 1986;51:1173–82. https://doi.org/10.1037/0022-3514.51.6.1173.
